# Turning evidence into recommendations: Protocol of a study guideline development groups

**DOI:** 10.1186/1748-5908-2-29

**Published:** 2007-09-05

**Authors:** Susan Michie, Jessica Berentson-Shaw, Stephen Pilling, Gene Feder, Paul Dieppe, Rosalind Raine, Francoise Cluzeau, Phil Alderson, Simon Ellis

**Affiliations:** 1 Health Services Research Collaboration, University of Bristol and Centre for Outcomes Research and Effectiveness, Department of Psychology, University College London, 1-19 Torrington Place, London WC1E 7HB, UK; 2 Centre for Outcomes Research and Effectiveness, Department of Psychology, University College London, 1-19 Torrington Place, London WC1E 7HB, UK; 3 Centre for health sciences, Institute of Health Sciences Education, Barts andthe London Queen Mary's School of Medicine and Dentistry, 2 Newark Street, London E1 2AT, UK; 4 MRC Health Services Research Collaboration, Department of Social Medicine, University of Bristol, Canynge Hall, Whiteladies Road, Bristol BS8 2PR, UK; 5 Department of Epidemiology & Public Health, University College London, 1-19 Torrington Place, London WC1E 6BT, UK; 6 Centre for Clinical Practice, National Institute for Health and Clinical Excellence, (NICE), MidCity Place, 71 High Holborn, London WC1V 6NA, UK; 7 Centre for Public Health Excellence, National Institute for Health and Clinical Excellence, (NICE), MidCity Place, 71 High Holborn, London WC1V 6NA, UK

## Abstract

**Background:**

Health care practice based on research evidence requires that evidence is synthesised, and that recommendations based on this evidence are implemented. It also requires an intermediate step: translating synthesised evidence into practice recommendations. There is considerable literature on evidence synthesis and implementation, but little on how guideline development groups (GDGs) produce recommendations. This is a complex process, with many influences on communication and decision-making, *e.g*., the quality of evidence, methods of presentation, practical/resource constraints, individual values, professional and scientific interests, social and psychological processes. To make this process more transparent and potentially effective, we need to understand these influences. Psychological theories of decision-making and social influence provide a framework for this understanding.

**Objectives:**

This study aims to investigate the processes by which GDGs formulate recommendations, drawing on psychological theories of decision-making and social influence. The findings will potentially inform the further evolution of GDG methods, such as choice of members and procedures for presenting evidence, conducting discussion and formulating recommendations.

**Methods:**

Longitudinal observation of the meetings of three National Institute of Health and Clinical Excellence (NICE) GDGs, one from each of acute, mental health and public health, will be tape recorded and transcribed. Interviews with a sample of GDG members at the beginning, middle, and end of the GDG's work will be recorded and transcribed. Site documents including relevant e-mail interchanges, GDG meeting minutes, and stakeholders' responses to the drafts of the recommendations will be collected. Data will be selected for analysis if they refer to either evidence or recommendations; the focus is on "hot spots", *e.g*., dilemmas, conflicts, and uncertainty. Data will be analysed thematically and by content analysis, drawing on psychological theories of decision-making and social influence.

## Background

Widespread claims of evidence-based clinical practice are premised on development of recommendations based on research evidence. Using research evidence to inform practice requires three stages: synthesising evidence, translating evidence into recommendations, and implementing recommendations in practice. There is considerable literature on the processes of synthesising evidence, and of implementing recommendations, but little on how evidence is translated into recommendations. The model of guideline development adopted by the National Institute for Health and Clinical Excellence (NICE) in the United Kingdom involves a Guideline Development Group (GDG) comprising academic, professional, and lay representatives from relevant disciplines and practices. Groups meet between three and fifteen times to consider evidence and recommendations. Their face-to-face and e-mail discussions are informed by: Verbal and written presentation of research evidence by systematic reviewers and health economists as well as contextual evidence from co-opted experts; comments on the evidence and draft recommendations by stakeholders; and comments and advice by NICE internal review teams.

Despite the stated evidence base of guideline recommendations, there is little information about the relationship between research evidence and recommendations. A study of 15 clinical guidelines on Type 2 diabetes from 13 countries found that research evidence is not necessarily the most powerful influence on the content of recommendations [1]. The investigators found little overlap between guidelines in the evidence used: only 18% of citations were shared with any other guideline, and only 1% appeared in six or more guidelines. Despite this, there was a high degree of consensus about recommendations about clinical care. Very different results were found in a study of two independent expert panels formulating appropriateness criteria for investigation of patients with angina. Given the same evidence summary and using a formal consensus process, the two groups showed only moderate agreement in their recommendations, with a two fold difference in their estimates of under-use for investigations in some subgroups of patients (Hemingway, *et al*., personal communication).

There is often no explicit framework for the conduct of the discussion or decision-making within GDGs. There are various possible influences on group discussion and decisions, *e.g*., the quality of evidence, the methods of presentation of the evidence, evaluation of different types of evidence, weight given to economic evidence, practical/resource constraints, opportunity costs, values (implications for inequalities), degree of change required by the recommendations, professional and scientific interests, potential direct or indirect financial interests [2,3], and the knowledge and experience of the group members in evidence evaluation and synthesis. Additionally, social and psychosocial processes within small groups (*e.g*. status, conformity and compliance) may influence guideline development [4]. Several tensions within this process have been reported: The extent to which recommendations should reflect contextual experience rather than just research evidence; the extent to which practical constraints should shape recommendations; and whether more evidence is needed to stop current practice than to start something new. A recent study using formal consensus methods for clinical guidelines found that half of group judgments did not agree with the research evidence. Where clinical experience and beliefs were not consistent with research evidence, the experience and beliefs seemed to take precedence [5].

There are calls for greater transparency about the processes of synthesising research and other evidence and of translating these into recommendations, but how best to do this is unclear. There are also calls for current practice to be changed to briefer, more structured deliberations [6]. However, changing future practice should be informed by understanding current practice.

Communication with experts in the field and subsequent literature searches identified four studies that have analysed GDG processes. A pilot study has examined normative influences on guideline construction, although it was unclear what were "normative influences" [7]. Pagliari and Grimshaw analysed social interaction between members and amount of communication according to professional status, and found more communication amongst higher status professionals [8]. A sociological analysis of the structure and organisation of group processes found four domains of group reasoning: the scientific quality of evidence, the practical basis of recommendations, political implications of recommendations, and the quality of the process that evaluated evidence and formulated recommendations [9,10].

The current study aims to build on this work by drawing on psychological theories of decision-making and social influence in describing and analysing how issues in these areas are resolved, with a goal of identifying good practice. It will do this across three health care settings (acute health, mental health and public health).

## Aims

To investigate the processes by which GDGs formulate recommendations on the basis of the research evidence they receive, using psychological theory to guide data analysis.

The main questions to be addressed concern:

1. Who? Who has most influence, demonstrates particular approaches

2. How? What strategies are used in considering evidence and in formulating recommendations

3. Why? What beliefs about the purpose and nature of evidence and recommendations may explain these strategies

4. With what result? GDG members views about the quality of GDG process and outcome; objective quality indicators of the recommendations, *e.g*., clarity, comprehensibility.

## Methods

### Study design

A longitudinal observation study of the development of national guideline practice recommendations, from the first to last GDG meeting.

### Participants

The first NICE GDG to start from November 2006 in each of three areas of health care, acute health, mental health and public health, will be selected. The GDGs participating in the study are not specified in order to protect anonymity of the members.

### Data collection

Data will be collected from several sources:

#### Meetings

Meetings of GDGs and their topic group will be tape recorded and transcribed. Using an ethnographic approach, a researcher will attend meetings, take field-notes, and make observations. Members will be asked to introduce themselves by name and role at the beginning of each meeting to assist in their identification. The researcher will position herself in a place that is as unobtrusive as possible, but that will allow identification of speakers and a view of members' facial expressions. Not all meeting recordings will be transcribed and analysed. A pre-validated process will be used to identify the key exchanges and interactions relating to recommendations and evidence. Data from the pilot study is being used to develop criteria for this process.

Site documents include relevant e-mail interchanges, GDG meeting minutes, and stakeholders' responses to draft recommendations. Given the large number of e-mails that each GDG may generate, the research group will use the pilot study to develop a "key word" search approach to filter the site documents on the basis of their relevance to evidence and recommendations.

#### Individual interviews

Following the first GDG meeting, the researcher will select a purposive sample for interviews from the different constituencies in the group (*i.e*., service providers, academics, practitioners, service users, systematic reviewers, NICE project team and chair). A selection of group members, representing different levels of involvement, will be interviewed at the beginning, middle, and end of the GDG process. Higher order interview questions have been developed to investigate GDG members' perceptions of the factors influencing their thinking and decisions about recommendations (additional file 1). These higher order questions will be mapped to specific incidences from each guideline meeting (identified by the researcher) in order to ensure the interviews are specific and focussed. Interviews will be audiotaped and transcribed.

#### Group interviews

The findings from the GDG discussions and interviews will be presented to GDGs for their comments to inform the face "validity" of the findings as well as any implications of the findings for future practice. The findings will be presented and responses sought first by e-mail to GDG members, followed by a discussion with the GDG as a whole. This will allow less vocal GDG members to input their views, while also enabling group interaction.

#### Stakeholders' comments

These are collected routinely and presented to the GDG.

Contextual influences may occur during the study period, *e.g*., high profile media or scientific publications. If these are discussed within the context of any GDG, they will be highlighted in the analysis.

### Piloting

The above procedures – gaining consent, the GDG observation, transcript analysis, collection and use of e-mail data – were piloted in September 2006 on a GDG that was considered by NICE members of the study steering group to be fairly representative.

### Data analysis

Data will be selected if they refer to either evidence or recommendations; the focus will be on "hot spots", *e.g*., dilemmas, conflicts, and uncertainty. Selected transcript excerpts will be analysed thematically and by content analysis. The analysis of the transcripts and other data will draw upon a number of different theories, including group decision-making [11,12] and communication/persuasion [13] theories. In consensual group decision-making (such as that occurring in NICE GDGs), it is assumed that there are background factors at play in the group's decision-making processes beyond the expression of mere individual preferences. These background factors include cognitive and affective components that are shared among the group during the decision-making process [11]. Such components include majority/plurality processes (the degree to which members' preferences about information and evidence are shared socially), group polarisation (processes by which extremes in an individual's preference will be exaggerated when the group's preferences are combined), procedural manipulation (the manipulation of group preferences presenting information in a specific order or manner), and the common knowledge effect (where shared information is more likely to be discussed within a group than unshared information). Specific features of communication and the process of persuasion may influence how GDGs translate evidence into recommendations. The elaboration likelihood model of persuasion [13] suggests that when individual group members are subject to persuasive messages from others in their group with specific agendas, their ability and desire to cognitively elaborate on that message may influence the process and outcomes of decisions about the evidence.

In data analyses, discrepancies between reported and observed processes will be highlighted, as will any areas of tension or difficulty. Case studies will be systematically identified and analysed within the development process, *e.g*., uncontroversial translation of evidence into recommendations; lack of evidence for a practice but recommendation for it; clear evidence but no associated recommendation made; mixed evidence and no recommendation; and mixed evidence and recommendation made.

A variety of published GDG recommendations will be used to develop a reliable measure of recommendation clarity, *e.g*., readability, perceived comprehensibility, specificity and avoidance of ambiguity, and building on prior research [14,15].

### Deliverables

The primary deliverable for the study will be a set of recommendations, including the composition of GDGs, the presentation of evidence, and discussion of recommendations to aid transparent decision-making and reporting in GDGs.

### Data management

A signed confidentiality agreement between the researchers, NICE, and the GDG chairs specify which GDG documents will be accessed, where they will be kept and reviewed, storage/disclosure, and access to data. Data will be anonymised as much as possible, and individuals will be given the opportunity to see draft reports to satisfy themselves about anonymity.

### Ethics

The study received ethics approval from the Research Ethics Committee of the UCL Psychology Department (ref: 0819/001). A study information sheet (additional file 2) will be provided to all GDG members before their first GDG meeting, and informed consent (additional file 3) will be sought for use of the following data: transcripts of GDG discussions, site documents (minutes arising out of the GDG meetings, e-mail exchanges about GDG business between GDG members, and draft versions of the recommendations), interviews with GDG members, and feedback focus groups of GDG members to comment on findings from the transcripts, e-mails, and interviews. Comments from non-consenting GDG members will not be transcribed.

## Competing interests

SM, JBS, PD and RR declare that they have no competing interests. GF has been paid to chair two NICE guideline development groups and one guideline development group by Weleda. FC, PA and SE advise on the methodology used for developing clinical guidelines at NICE.

## Authors' contributions

SM conceived and developed the study, drafted the study protocol, and leads its implementation. SM helped to draft the manuscript. JBS coordinates the ongoing study, collected and analysed pilot data, and helped to draft the manuscript. SP, GF, PD, RR, FC, PA, and SE are members of the Study Steering Group, and have contributed to the development of the protocol. All authors read and approved the final manuscript.

## Supplementary Material

Additional file 1 Draft Interview Questions. These are the core questions that were adapted for interviews with Guideline Development Group members. Click here for file

Additional file 2 Study Information Sheet. This is the information sheet about the study given to eligible participants. Click here for file

Additional file 3 Study Consent Form. This is the consent form that participants signed. Click here for file
