# Supplementary material for: Turning evidence into recommendations: Protocol of a study guideline development groups
Source: Implement Sci. 2007 Sep 5;2:29. doi: 10.1186/1748-5908-2-29 (PMC2031892; doi:10.1186/1748-5908-2-29)
Supplement: Additional file 1 — Draft Interview Questions. These are the core questions that were adapted for interviews with Guideline Development Group members. [file 1748-5908-2-29-S1.pdf]

**Appendix A. Draft Interview Questions**

| | |
|---|---|
| 1 | Can you describe what you think is the process through which recommendations are decided in your GDG? |
| 2 | What factors do you take into account when making/deciding on a recommendation? How do you decide the weight to give these factors? |
| 3 | Do you think that other members of the GDG take *different* factors into account when making a recommendation and if so what would these be? |
| 4 | What do you understand the word 'evidence' to be referring to in the GD process? |
| 5 | Do you think your understanding and use of the word 'evidence' is different from other members of the group? If so how do these differences influence or affect the recommendations that are developed by the group? |
| 6 | Does tension arise in the group as a result of discussions about 'evidence'? If so can you describe what the tension is about with regard to the 'evidence', and why you think it arises? |
| 7 | What if anything is done to resolve the tension in the group resulting from discussions of the 'evidence'? |
| 8 | If such tensions do arise does it affect how you feel about your or other's role in the group? |
| 9 | When formulating a recommendation how do you think the group evaluates the importance of different types of 'evidence'? |
| 10 | What do you think the purposes of recommendations are? |
| 11 | Do you ever think that the evidence suggests recommendations that would be impossible or very difficult to implement? If so, why? |
