# Supplementary material for: Turning evidence into recommendations: Protocol of a study guideline development groups
Source: Implement Sci. 2007 Sep 5;2:29. doi: 10.1186/1748-5908-2-29 (PMC2031892; doi:10.1186/1748-5908-2-29)
Supplement: Additional file 2 — Study Information Sheet. This is the information sheet about the study given to eligible participants. [file 1748-5908-2-29-S2.pdf]

Appendix B: Study Information Sheet

CENTRE FOR OUTCOMES RESEARCH AND EFFECTIVENESS
UCL

UCL

"Turning Evidence into Recommendations"

You are invited to take part in a study investigating how guideline development groups (GDGs) develop recommendations from the research evidence. The project, run by the Centre for Outcomes Research Effectiveness (CORE) at University College London, will study three GDGs preparing guidance on behalf of the National Institute for Health and Clinical Excellence (NICE) over the next year.

What is the purpose of the study?
The study aims to describe how research evidence is used in formulating recommendations by GDGs, with a view to improving future GDG practice, and to provide a framework for transparent decision making and reporting.

Do I have to take part?
Participation is voluntary. Please take time to consider whether you would be willing to participate. The study may have important implications for how guidelines are developed in the future.

What will participation in the pilot study involve?
Participation will involve the following:

1. All key meetings of the GDG will be observed, taped and analysed by a researcher.
2. The researcher will read some of the guideline relevant e-mail correspondence between GDG members (we want to stress we will only look at those emails that refer directly to guideline business).
3. You may be interviewed by a researcher trained in conducting interviews, who will ask about your experience of participation in a GDG; the interviews will be recorded. You will not be under any obligation to answer any questions you do not wish to.

You will be free to withdraw from the study at any point, practically speaking this will mean that although your comments will still be recorded in the GDG meetings, they will not be transcribed or analysed, your emails will not be examined and you will not be interviewed.

Will my taking part be kept confidential?
The information that you provide will be kept strictly confidential. The information will be stored using study numbers, and your name and any other identifying information will not be used. Interview tapes and transcripts will be stored securely at CORE, and will be destroyed after the study. Only the research team will have access to them. Excerpts of transcripts may be included in any potential publications or presentations arising from the study, but we will anonymise these comments and any publications will be shared with you at draft stage and you will have the right of veto over excerpts that you feel contains information that might identify you.

Who is organising and funding the research?
The project is led by Susan Michie, Professor of Health Psychology from University College London, and is a joint collaboration with NICE. It is funded by the Medical Research Council.

Who has reviewed the study?
This research was approved by the Research Ethics Committee at UCL's Psychology Department.

For further information regarding the project, please contact:
Susan Michie, Professor of Health Psychology, Centre for Outcomes Research and Effectiveness, UCL and MRC Health Services Research Collaboration, University of Bristol.
s.michie@ucl.ac.uk
