# Supplementary material for: Turning evidence into recommendations: Protocol of a study guideline development groups
Source: Implement Sci. 2007 Sep 5;2:29. doi: 10.1186/1748-5908-2-29 (PMC2031892; doi:10.1186/1748-5908-2-29)
Supplement: Additional file 3 — Study Consent Form. This is the consent form that participants signed. [file 1748-5908-2-29-S3.pdf]

**Appendix C: Study Consent Form**

**CENTRE FOR OUTCOMES RESEARCH AND EFFECTIVENESS**
**UCL**

**EVIDENCE INTO RECOMMENDATIONS**

Name of Researcher: Prof Susan Michie

***Please read each statement and tick the box if you agree***

1. I confirm that I have read and understand the information sheet dated ……………………… for the above study. I have had the opportunity to consider the information, ask questions and have had these answered satisfactorily. ☐
2. I understand that my participation is voluntary and that I am free to withdraw at any time, without giving any reason, without my legal rights being affected. ☐
3. I understand that the meetings I attend will be recorded and transcribed and excerpts may be looked at by responsible individuals from the research team. I give permission for the research team to have access to the data. ☐
4. I understand that I may be interviewed about the guideline group process and the transcript of this interview may be looked at by responsible individuals from the research team. I give permission for the research team to have access to the data. ☐
5. I understand that email correspondence relating to the guideline group I am part of may be collected during the study and may be looked at by responsible individuals from the research team. I give permission for the research team to have access to the data. ☐
6. I agree to take part in the above study. ☐

| | | |
|---|---|---|
| ________________________ | ________________ | ____________________ |
| Name of Participant | Date | Signature |
| ________________________ | ________________ | ____________________ |
| Researcher | Date | Signature |

When completed, 1 for participant; 1 for researcher site file; 1 for National Collaborating Centre files
